# Evaluation of interprofessional student teams in the emergency department: opportunities and challenges

**DOI:** 10.1186/s12909-022-03954-y

**Published:** 2022-12-19

**Authors:** Kerry Hood, Wendy M. Cross, Robyn Cant

**Affiliations:** Institute of Health and Wellbeing, Federation University Australia, PO Box 859, Berwick VIC 3806, 100 Clyde Rd, Berwick, Victoria Australia

**Keywords:** Emergency department, Interprofessional education, Program evaluation, Self efficacy, Students, medical, Students, nursing

## Abstract

**Background:**

Interprofessional education opportunities are commonly university-based and require further development during clinical practice. Many clinical contexts offer the potential for meaningful learning of both collaborative and discipline-specific practice. The emergency department (ED) demands efficient teamwork, so presents a logical location for interprofessional learning.

**Methods:**

An interprofessional clinical placement program was implemented with the aim to enhance students’ capacity and self-efficacy for collaborative practice. Fifty-five medical and nursing students participated as interdisciplinary pairs in a two-week clinical placement in the ED. Students’ perceptions of the learning environment were measured pre- and post-placement with the Self-efficacy for Interprofessional Experiential Learning Scale and the Interprofessional Clinical Placement Learning Inventory was completed post-placement. Non-parametric tests were used to establish change differences.

**Results:**

The Placement Learning Inventory revealed positive outcomes; the majority (16/19) agreed/agreed strongly that the placement provided sufficient learning opportunities, was interesting, and made them feel as if they belonged and most (14/19) reported they achieved the discipline specific learning objectives set by the university. Self-efficacy improved significantly (*p* = 0.017), showing promise for future use of the placement model Challenges were identified in the organisation and supervision of students. In the absence of additional dedicated student supervision, this model of interprofessional student pairs in the ED was challenging.

**Conclusions:**

Interprofessional clinical placements in ED are an effective clinical learning approach for final year undergraduate medicine and nursing students. Recommendations for improvements for students’ clinical supervision are proposed for the placement model.

**Supplementary Information:**

The online version contains supplementary material available at 10.1186/s12909-022-03954-y.

## Introduction

Interprofessional education (IPE) has been proposed as a mechanism to prepare health professional students for collaborative practice [1, 2]. Despite the repeated calls over many years to further develop opportunities for IPE in practice, IPE remains typically university based [3–5]. A review of literature on practice-based interprofessional learning identified multiple education models, varying from 2-hour workshops and case studies to one-day simulations and group sessions [6]. According to O’Leary et al. [5], a minimum duration of 2 weeks is recommended for meaningful impact on future practice. Longer 2-week interprofessional training wards (IPTW) have remained the most sustainable model of practice-based IPE [7] when organisational infrastructure is well-established [5].

Interprofessional training wards are effective environments for pre-registration healthcare learners because they offer authentic rehearsal of professional roles [8]. IPTWs have been operational in Scandinavia for many years, commonly in orthopaedic, medical and aged care wards [7] with some translation of the model reported in Australia [8, 9] and more recently in Germany [10, 11]. IPTWs have also been trialled in the emergency department (ED) with some success [12] although are yet to be translated to the Australian ED context.

A retrospective review of 7 years of a program in Sweden explored IPTW student feedback and reported medical, nurse, physiotherapy, and occupational therapy students’ perspectives on the processes of learning in an orthopaedic ward [13]. The review reported that the IPTW provided an enriching learning environment with authentic and relevant patients, well-composed and functioning student teams, competent and supportive supervisors, and adjusted ward structures to support learning. In addition, students improved awareness of their own development with belief in their ability to practice in the future, through rehearsing their future roles [13]. Similar findings are reported by Pelling and colleagues in their 5-year review of an orthopaedic IPTW [14]. They found that students’ understanding of their own and others’ roles improved, along with their valuing of teamwork. The pioneers of the Linkoping model have reported more than two decades of successful IPTW experience, and this program has been sustained [15].

A key contemporary view of the field that confirms these assessments is seen in a recent review of 37 studies of interprofessional training wards in 12 different institutions [16]. The IPTWs involving multiple professions (usually in teams of between two and 12 students and over a period of 2 weeks) showed promising results with regard to short-term student learning outcomes and patient satisfaction rates. Therefore, it is possible to conclude that the overall literature supports positive outcomes and that the IPTW model is acceptable and effective for student education. Practice-based IPE helps to prepare students for collaborative practice and to be ready for entry to the healthcare workforce.

Healthcare faculties are tasked with producing ‘work-ready’ graduates, yet the literature continues to indicate short-comings among graduates [17, 18]. Social intelligence, communication and teamwork skills are presented as critical features of work readiness [18]. Self-efficacy is an important concept in preparing students for the workforce; self-efficacy reflects an individual’s beliefs in their competence and confidence to take on tasks and their perseverance in the face of challenge [19]. Healthcare systems are team based, complex, and depend on healthcare professionals with sophisticated levels of interpersonal communication and collaborative skills. The stakes are high for newly graduated health professionals, the organizations they work in, and most importantly, the patients they treat. A lack of teamwork and poor communication are consistently among the top contributors to sentinel events, with over half a million adverse events annually in Australian hospitals alone between 2012 and 2018 [20]. Improving the confidence, or self-efficacy, of future health professionals to better collaborate and communicate may help to avoid preventable errors and improve patient safety.

We conducted an interprofessional clinical placement program in the ED. The student placement was based on the long-standing model of the interprofessional training ward pioneered in Sweden in the 1990s [21, 22]. This new intervention was an evolution of an earlier model tested in this ED, reported previously by Meek et al. in 2013 [23]. The original study found the IP student placement model valid for application in ED, with ED performance indicators for patient throughput and the quality of care being maintained for patients managed by either students or clinicians [23]. Further investigation was needed to determine whether an adapted model, where student teams worked alongside and were supervised by clinicians, remained reasonable and appropriate for their learning.

## Methods

### Aim

The study aimed to investigate the outcomes of a program of interprofessional clinical placements in a metropolitan hospital ED. The purpose of the clinical placement was to enhance students’ capability and self-efficacy for collaborative practice.

### The intervention

The interprofessional clinical placement program in the ED was designed in consultation with international and local experts in interprofessional education, university staff and health service clinicians. The model has final year (3rd year) nursing students and final (5th) year medical students working as a paired team alongside clinicians in a hospital ED over a period of 2 weeks (10 working days) on day shifts. All students who had been allocated to ED clinical placement by their university over the IPL period were invited to participate in the interprofessional experience by expressing interest.

The ED, in a metropolitan tertiary hospital in the state of Victoria, Australia, managed over 47,000 patient admissions annually. A limited number of beds were deemed appropriate and available for student care provision by two student pairs, which formed the main reason for the student sample being small.

Additionally, each placement duration was 2 weeks with one student pair, thus limiting the overall number of student participants. Interested students were selected on the basis of the dates of their placement coinciding with the IPL program over 16 weeks. (No record was kept of how many applicants were unsuccessful). Other students remained on placement in ED in the traditional placement model.

Participating students were provided with learning material and information about the interprofessional placement prior to commencing in the ED. They completed a half-day orientation program on day 1 of the IP placement.

**Fig. 1:**
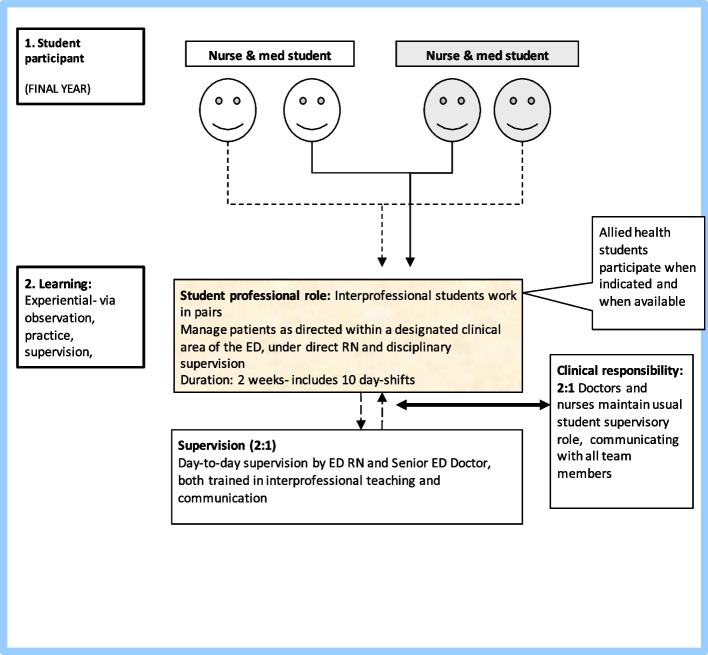
Work-flow chart – A model for interprofessional training ward placements based on nursing and medicine final year undergraduate student teams

Over eight placement fortnights in 2016 and 2017, 26 nursing students and 29 medical students completed the program as dedicated paired student teams. During the first half of the project, five pharmacy students and four physiotherapy students participated in the student teams for a single day placement. This was discontinued in the second half due to the difficulties with differing placement models and supervision requirements for these students. The student workflow is depicted in Fig. 1.

#### Students’ interprofessional role

Students worked within the boundaries of their scope of practice, supervised by an experienced ED nurse and a doctor who were trained in facilitating interprofessional learning. Students were allocated selected patients with lower levels of acuity who were situated in one set of ED bays comprised of four beds, consistent with the nursing and medical workload allocation of the ED. Under supervision, student teams conducted all aspects of patient care including assessment, decision making about tests and investigations, planning of patient management and implementation of care, from patient admission to hospital, to their discharge or transfer. There was a maximum of two parallel medical/nursing student teams, that is four students per shift. It was not known what previous experience of interprofessional education had been completed by the placement students, as some university-and health service based short seminar programs were offered but were not compulsory.

Training for clinical supervisors involved a three-hour workshop about interprofessional facilitation, which blended individual preparation and a face-to-face interactive session. The learning objectives of the training workshop included: competence in interprofessional communication, team-working and collaboration and knowledge of other’s professional roles and boundaries. All supervisors had access to an online resource on the university’s learning management system, which held interprofessional training materials and facilitator resources. Interprofessional facilitator training is recommended for all supervisory clinical staff when establishing interprofessional learning [12, 24] and had been confirmed as good practice in a prior trial related to this study [25]. Students were allocated to clinical shifts alongside trained IP facilitators. The clinical supervision for student teams over the 10 days of a placement could, therefore, involve a number of different supervisors from each profession.

#### Evaluation

A survey approach was utilized for evaluation of this study. At the level of the organization, we explored the ED context as a site for interprofessional learning. At the level of the individual student, we explored student learning and self-efficacy in collaborative practice.

The research questions to be answered were, therefore:

(i) How effective is the ED as a location for an interprofessional training ward for final year students?
(ii) Does student self-efficacy improve after a two-week interprofessional placement?

#### Measurement instruments

The Self-efficacy for Interprofessional Experiential Learning Scale (SEIES) was used to measure students’ self-confidence in their ability to take on tasks and to persevere despite barriers they may encounter (RQ2). The SEIES instrument was selected as most appropriate to the placement context and interprofessional roles to respond to RQ2, as very few scales explore the specific contexts. Developed by Mann and colleagues [26], this 16-item and 10-point scale (1 = low confidence, 10 = high confidence) showed good internal validity for the scale (α = 0.96) and two subscales (Cronbach’s α = 0.94 and 0.93 respectively) when tested with 209 Canadian healthcare students. This scale was completed by participating students as an initial pre-test and a post-test with students allocating a personal code so that repeated surveys could be paired.

The student evaluation surveys were paper-based and collected anonymously in a ward post-box.

At the end of the placement, students completed a course evaluation survey: Interprofessional Clinical Placement Learning Inventory (ICPLEI) (RQ1), provided as Additional File 1 [see Additional File 1]. This 26-item, five-point scale was developed by members of the research team in a previous study to measure healthcare students’ perceptions of an interprofessional clinical placement experience, including orientation, supervision, roles, learning and autonomy [27]. The scale’s reliability was confirmed with Australian nursing, medical and allied health students (*n* = 38), a Cronbach alpha of 0.80 and moderate item-to-total correlations for 22/26 items. The Cronbach alpha with the current sample was adequate (α = 0.81). The survey included three open-text options asking about the best/ worst aspects of the course and suggestions for course improvement. The reasons for selecting this instrument is that it relates to the specific context under investigation, and has been trialled and found valid with similar IPL cohorts [6, 27].

### Data collection

Students provided written consent to participate in the research. The SEIES was completed by participating students as an initial pre-test and a post-test with students allocating a personal code so that repeated surveys could be paired. On the last day of the placement students completed the placement evaluation inventory (ICPLEI).

The student evaluation surveys were paper-based and were collected anonymously in a ward post-box.

### Data analysis

Descriptive and summary statistics (means, medians, standard deviations) were computed for quantitative data to report scale results using IBM-SPSS Statistics for Windows Vs 25 (Armonk, NY: IBM Corp.). Between group differences were explored using non-parametric statistical tests (e.g., the Mann Whitney U Test for independent samples and Wilcoxon Signed Ranks test for paired samples). *P* < 0.05 was regarded as significant. Missing data were not replaced, and this reduced the overall sample.

To explore the difference across student professions, student’s’ responses in open questions within the ICPLEI were mapped in a Word document to show medical and nursing discipline textual responses to explore the difference across student professions. Following the thematic analysis method of Creswell and Clark [28] the two sets of student responses were read and re-read by two researchers. Two researchers used open coding to tabulate and cluster response text to develop an understanding of program features and map those that were applicable to students in each profession. Further discussion and integration of these data sources with the agreement of both researchers enabled key themes to be generated.

## Results

We present the results in response to the two research questions.

### **RQ1:** how effective is the ED as a location for an interprofessional training ward for final year students?

Nineteen students (seven medicine, 12 nursing) gave their perspectives about the effectiveness of the ED as a location for interprofessional learning. This equates to a response of 34.5% (19 of 55). The student responses are presented in Table 1 below.

**Table 1:** Student responses to the IPCLEI (*n* = 19)

Item	Mean/SD
1. The purpose (learning objectives) of this placement was made clear	4.32 (0.75)
*2. I need more orientation to this placement*	*2.74 (1.01)*
3. Orientation was relevant and well organized	4.05 (0.71)
4. The teaching strategies helped my learning	3.63 (0.76)
5. My preference is for the teachers to be of the same discipline as the student	3.79 (0.98)
6. I valued having more than my own discipline being involved in teaching	4.00 (0.88)
*7. There was too much supervision in this placement*	*2.58 (1.07)*
8. This clinical placement was interesting	4.05 (0.91)
*9. The workload was too heavy*	*2.21 (0.63)*
*10. There was too much pressure on me in this placement*	*2.26 (0.87)*
11. This clinical placement was well organized	3.53 (0.07)
12. I usually had a clear idea of what was expected of me	3.84 (0.76)
13. I achieved the discipline specific learning objectives set by my university	3.84 (0.76)
14. My other student commitments did not interfere with my involvement in this placement	3.63 (1.01)
15. The placement provided me with sufficient learning opportunities	4.11 (0.81)
16. I felt as if I belonged to the ward	3.84 (1.21)
17. The teachers were friendly and approachable	4.32 (0.58)
18. This placement has given me new insights into how a ward is run and managed	4.26 (0.81)
19. After this placement I understand more fully my discipline’s role in the IP clinical team	4.05 (0.71)
20. After this placement I have a greater understanding of the role and function of other disciplines in health care	4.26 (0.56)
21. I felt comfortable in asking for advice or assistance when necessary from my student colleagues	4.58 (0.61)
*22. I felt uncomfortable taking the lead in a student group*	*2.37 (0.89)*
*23. I felt uncomfortable sharing responsibility for delivery of health care*	*1.89 (0.57)*
24. I felt comfortable putting forward my opinions in a group	4.11 (0.57)
25. After this placement I have a better understanding of the patient’s role in healthcare decision-making	4.05 (0.85)
26. I felt comfortable communicating with patients and their families to seek their input into care	4.42 (0.51)

NOTE: Ratings from 1 (strongly disagree) to 5 (strongly agree). Six negatively worded questions are highlighted with italics, however these indicate positive support for the program

There was strong support for learning in the ED context because it was an opportunity for application of the students’ knowledge and rehearsal of behaviours that would be needed in their future roles. Based on agreement with statements in the ICPLEI, many students (16/19) agreed/agreed strongly that the placement provided sufficient learning opportunities, was interesting, and it made them feel as if they belonged to the ward. The majority (14/19) reported they achieved the discipline specific learning objectives set by the university. The most common comment from students was that this was their first experience of independent practice, and they appreciated the “level of independent practice” that was permitted.

Eighteen of 19 students who responded to the ICPLEI agreed/strongly agreed that they developed a greater understanding of the role and function of other disciplines in health care and 15/19 agreed/strongly agreed they had a better understanding of the patient’s role in healthcare decision-making.

The interprofessional placement in ED was also perceived as beneficial in enabling a broader understanding of students’ future professional roles. Almost all (17/19) agreed/agreed strongly that the ED placement gave them a greater understanding of the role and function of other professions and gave new insights into how a ward is run and managed.

Additionally, most (14/19) felt comfortable in asking for advice or assistance when necessary, from student colleagues and valued having disciplines other than their own involved in teaching and learning. Open text comment supported these views. The best aspects were:

Learning to communicate in a team and collaboratively manage care (nursing student).

Teaming up and appreciating the nursing role in patient care (medical student).

Working in IPL meant that I got to be a teacher as well as a student (nursing student).

Getting first-hand experience of the nursing world, learning from my colleagues and growing together as the placement progressed (medical student).

There were, however, comments from medicine and nursing students about the nature and consistency of clinical supervision. Students were disappointed at a lack of continuity in supervisors. Some nursing students perceived they only had medical supervision, with the resulting view that a medical focus was sometimes dominant. This was despite students being allocated to both medical and nursing supervisors on every shift. This warrants further investigation.

Some student comments about the organisational and supervision problems are described below:

I found there were inefficiencies in patient care when always having to wait for both nursing and medical supervisors to get things done (medical student).

Some days … were a little bit slow, hard to find nurses for help and spent long waiting periods waiting for things to happen (nursing student).

Supervisors were variable in training for IPL. Suggest increased awareness & requirement for training (medical student).

I felt that at times the IPL placement was slightly disorganised, many staff members were asking me how it worked (nursing student).

### RQ2: does student self-efficacy improve with a two-week interprofessional placement?

Initial self-reported confidence in ability (self-efficacy) surveys (SEIES) were received from 15 nursing and 14 medicine students (53% response), while 12 nursing and 12 medicine students (43.6%) returned post-test self-efficacy surveys. Students were positive about gains in learning and improvements in self-efficacy that were achieved after participating in the interprofessional placement. In addition to their reports of learning clinical practices and achieving uni-professional learning objectives set by the university, the interprofessional nature of clinical supervision was seen as valuable.

Self- efficacy ratings are presented in Table 2. There was a strongly significant increase in self-efficacy ratings at the end of placement. Of a possible score of 160, the median initial score was 99 (62%) and post-test median rating was 128 points (80%) (z = − 2.83, *p* = 0.017). A paired sample showed a large effect size of *r* = 0.62 (using Cohen’s *d*). When responses were examined by profession (medicine, nursing), a non-significant difference was found between groups in pre-test or post-test total scores. Although nurses initially rated their confidence on average higher than medical students (*N* = 102 versus M = 93 respectively), this difference did not reach a level of significance. The greatest improvements were in the items: ‘Interacting with students from other professions and disciplines than my own’; ‘Understanding and discussing the objectives of interprofessional learning’; and ‘Communicating effectively with other members of an interprofessional team’. These results confirm that student outcomes include their engagement with a new concept: collaborative working.

**Table 2:** Students’ responses to the Self-efficacy for Interprofessional Experiential Learning Scale

	Medicine		Nursing	
	Pre test (Md) *n* = 14	Post-test (Md) *n* = 12	Pre-test (Md) *n* = 15	Post-test (Md) *N* = 12
1. Working with other students from different professions to form a team	6	8	7	8
2. Working with other students from different professions to resolve problems in the team.	6	7	6	7.5
3. Working with other students from different professions to develop a realistic appropriate patient care plan	6	7	6	8
4. Working with other students from different professions to understand our respective roles in an interprofessional team.	6	7	7	8.5
5. Working with other students from different professions to understand the benefits to patients of team care.	6	8	7	9
6. Understanding and discussing the objectives of interprofessional learning	6	9	7	9
7. Interacting with students from other professions and disciplines than my own.	7	9	7	9
8. Providing feedback to an interprofessional team on our function and work as a team.	6	8	6	8.5
9. Providing feedback to individual team members of an interprofessional team on their function and work on the team	6	8	6	8
10. Helping clinical sites understand an interprofessional team’s role in a clinical setting.	6	8	6	6.5
11. Helping the patient to understand the objectives of the interprofessional learning.	6	8	6	8
12. Evaluating the quality of the work as an interprofessional team	6	8	6	7.5
13. Evaluating the degree to which an interprofessional team has achieved its goals.	6	7	6	8.5
14. Learning together cooperatively with students from other professions	7	9	7	8.5
15. Communicating effectively with other members of an interprofessional team	7	7	6	9.5
16. Interacting with teachers and preceptors from other professions and disciplines than my own.	6	7	6	8.5

Note: Response scale on self-efficacy beliefs: 1 (low confidence in ability) to 10 (high confidence in ability)

## Discussion

This study evaluated an interprofessional placement in the ED which was developed in consultation with academic interprofessional experts, university leadership and health service clinical and education staff. Ensuring clinical outcomes and engagement of stakeholders remained a priority consideration, as has been recommended [29].

The ED is a complex clinical environment for learners. Clinical care is high stakes and fast-paced; patient acuity is highly variable. We had previously tested an interprofessional clinical placement model in this ED with positive outcomes for students and patients [23]. We used our past experience to fine-tune the model in consultation with key internal stakeholders and advice from international experts.

The results show that students perceived the ED as an effective environment for learning interprofessional skills and behaviours. Central aspects of team working were learned as the students worked in their pairs to manage real patients, as evidenced by student self-reporting and supervisor observations. These included improved ways of team working with colleagues, especially team communication and cooperation. Importantly, students provided direct care to patients in the ED within the clinical learning environment.

It has been suggested that students exposed to authentic teamworking in real clinical settings will develop positive attitudes towards interprofessional behaviour [30] and these findings support that position. It is noteworthy that, while not the focus of this study, students also completed their discipline-specific learning requirements during the placement and reported that they were excited to learn new clinical skills such as intravenous cannulation and the application of plaster casts. The finding that an interprofessional placement can meet both uni-professional requirements and interprofessional objectives is important. This suggests it may not be necessary to source additional clinical placement time, but that existing placements can be adapted for interprofessional learning. Collaboration in practice is the norm. Placement structures that embody the ideal of collaborative care in real clinical settings, as described by Miller and Paradis [31] can be instituted as evidenced in this study. Reframing clinical placements, or aspects thereof, will allow students to experience the realities and the possibilities for their own future roles in collaborative clinical practice. In addition, observing genuine collaboration in practice provides students with powerful role models and the perception that teamwork is the dominant norm - as has been suggested [30, 31].

A lack of consistency in approaches to supervision among professional staff was found to be an issue that compromised students’ satisfaction. The student supervisors in the current study were not supernumerary to the staffing of the ED and simultaneously held responsibilities for clinical care. While this is the usual manner of student supervision, supporting the IPTW structure was found to be challenging at times. The rotating roster of supervisors was a concern for students owing to different supervisor approaches to teaching and supervision of the student teams. On every shift, trained clinical supervisors were allocated to the student pairs. Despite this, students at times reported the lack of an available supervisor or perceived that they had to wait to consult with supervisors when they became available. Similar findings have been reported in a well-established IPTW in an ED [12].

Increased consistency in supervision, and thus feedback, over placement days may enhance the student team’s learning trajectory. In the previous iteration of the model in this ED [23], a dedicated nurse facilitator was responsible for the supervision and coordination of the student teams. This model of a focused support person who is not responsible for clinical care is worth revisiting, supported by a recent qualitative meta-synthesis finding that additional human resources are required for the success of interprofessional practice-based placements [5].

The logistical challenges associated with this study are nothing new. Discipline specific placement models, curriculum requirements and different shift commencement times for nursing and medicine were evident. Some nursing students felt the placement did not reflect a real model of nursing, suggesting a lack of understanding of the purpose of the placement. Clearer expectations about the nature and purpose of the interprofessional placement may assist in enabling a broader view of teamwork as clinically authentic. We were unable to negotiate a common start and finish time for the students’ clinical shifts, with nursing students commencing at 0700 and medical students commencing at 0800 as was usual practice for their professions. For future placements, we recommend negotiation to achieve agreement on common placement rosters for student and supervisor teams. This may assist with teamwork formation and maximise collaboration within a unified placement structure.

The finding of a significant impact of the placement on preplacement student self-efficacy scores suggests that the interprofessional placement experience was effective in increasing the likelihood of future interprofessional behaviour. This increase is an important finding as students with stronger self-efficacy may be more inclined to sustain interprofessional behaviours in the future. We need to better understand why interprofessional learning changes behaviour, or the likelihood of future collaborative behaviour [12, 32–34]. Future research examining self-efficacy and collaborative intention among health professional students is strongly recommended using a framework such as the Theory of Planned Behaviour [35]. The inclusion of social scientists to tackle the underlying complex issues of identity, social influence and social constructs is an important consideration as our understanding of interprofessionalism evolves [36, 37].

Both the interprofessional practice placement and the corresponding evaluation had limitations. These include: a small number of student pairs available during dates assigned for placement and student preference for the same supervisor throughout the placement. This raises an issue that the results may have limited generalizability, thus should be interpreted with some caution. Our results align with those of a recent review of practice-based IPE that highlights the need for more robust theoretical foundations, layered leadership, and a significant shift in placement culture [5]. The sustainability of the future IPTW experiences in the ED will be influenced by each of these attributes.

## Conclusion

The ED provides a natural environment in which to learn teamworking, however the optimal placement and supervision models require further investigation. This program of interprofessional placement was found to be beneficial by medical students and nursing students for learning to work alongside their colleagues, and lessons for future improvement of the placement model were gained. Whilst the program was feasible, further attention to the specific facilitators and barriers to IP training in the ED is required to embed a sustainable IPTW model.

Student self-efficacy gains following an immersive interprofessional placement were a key finding and should be further explored. We recommend that future interprofessional clinical learning is underpinned by educational and behaviour change theories in design and evaluation and should include experts in interprofessional education and social science. Future success will require robust relationships between education providers, health services and interprofessional researchers with the will to facilitate and sustain this model of ideal preparation of the future health workforce.

## Supplementary Information

**Additional file 1.**

## Data Availability

The datasets analysed during the current study are not publicly available due to university confidentiality arrangements but are available from the corresponding author on reasonable request.
